# Effect of Beta-Alanine Supplementation on Exercise-Induced Cell Damage and Lactate Accumulation in Female Basketball Players: A Randomized, Double-Blind Study

**DOI:** 10.2478/hukin-2022-0034

**Published:** 2022-09-08

**Authors:** Farhad Gholami, Ajmol Ali, Ali Hasani, Afsaneh Zarei

**Affiliations:** 1 Faculty of Sport Sciences, Shahrood University of Technology, Shahrood, Semnan, Iran; 2 School of Sport, Exercise and Nutrition, Massey University, Auckland, New Zealand

**Keywords:** buffering capacity, carnosine, oxidative damage, muscle damage

## Abstract

Beta-alanine (BA) is a supplement that has received attention for its buffering potential among athletes. The aim of this study was to investigate the effects of BA supplementation on exercise performance and exercise-induced cell damage in female basketball players. Twenty-two female basketball players participated in a randomized, double-blind study. They ingested 6.4 g·day^-1^ of BA or an isocaloric placebo (dextrose) over 4 weeks. Exercise performance including aerobic (Bruce test), anaerobic (Wingate test), intermittent (Yo-Yo test) and basketball performance (countermovement jump and free throw shots) was measured before and following the intervention. Exercise measures were performed at the lab and free throw shots were undertaken on a wooden indoor basketball court. Blood samples were also collected before and after the exhaustive exercise to assess lactate concentration, creatine kinase (CK), lactate dehydrogenase (LDH) and malondialdehyde (MDA) activity. The exhaustive exercise test induced an increase in lactate concentration and MDA, CK and LDH activity (all p < 0.05). BA supplementation significantly reduced the lactate response to exhaustive exercise (p = 0.001); however, it had no significant effect on exercise-induced MDA, CK and LDH activity (all p > 0.05). Furthermore, exercise performance measures improved from pre- to post-test regardless of supplement/placebo ingestion (all p < 0.05). BA consumption over 4 weeks significantly reduced lactate accumulation following exhaustive exercise, but had no ergogenic effect in female basketball players. Usual dosing of BA does not seem to exhibit protective effect against oxidative damage.

## Introduction

Basketball is a team sport game consisting of repeated high-intensity efforts interspersed with low-to-moderate-intensity recovery periods. Based on the characteristics of this sport, significant contribution of the anaerobic energy systems is required to maintain high-intensity exercise intervals during training. The high demand on anaerobic energy supply results in accumulation of H^+^ ions and decreased muscle and blood pH levels. Reduction in pH is linked to fatigue and performance impairment (Bishop et al., 2004) including technical tasks (Lyons et al., 2006; Bjelica et al., 2020 ) and free throws (Padulo et al., 2015). Fatigue induced by high-intensity exercise is detrimental to technical performance of novice and professional basketball players (Lyons et al., 2006). Thus, athletes seek to improve sports performance by utilizing various sports supplements.

Beta-alanine (BA) is a non-proteinogenic amino acid and the rate-limiting precursor of carnosine, which has intramuscular buffering potential. Indeed, the ergogenicity of BA lies with its potential in elevating muscle carnosine concentration. It has been reported that 3-6 g of BA ingestion over at least 4 weeks can increase intracellular carnosine content (Baguet et al., 2009; Derave et al., 2007). Elevated muscle carnosine may increase muscle buffering capacity and improve exercise performance, especially in events with significant energy supply from anaerobic glycolysis. However, there is discrepancy in the literature regarding ergogenic effects of BA in athletes (Baguet et al., 2009; Derave et al., 2007; Ducker et al., 2013) which may be due to several factors including the fitness level of participants, exercise modality, as well as the dose and duration of supplementation. For instance, BA consumption over 4 weeks improved total work performed on a cycle ergometer in male participants (Hill et al., 2007), whereas there was little ergogenic effect on swimming performance in a real-world setting (Chung et al., 2012). Moreover, 7 g·day^-1^ of BA over 28 days had no effect on 2,000 m rowing performance in well-trained male rowers (Ducker et al., 2013).

Despite numerous studies investigating ergogenic effects of BA on exercise capacity, little is known about ergogenicity of BA on sport-specific performance in team sport players. Besides, most studies have focused on male athletes and less research has investigated ergogenic potential of BA in female athletes. Females have been reported to have naturally lower intramuscular carnosine content compared with males and may experience a higher relative increase in carnosine levels following BA consumption (Everaert et al., 2011; Stegen et al., 2014). Thus, it is likely that female athletes respond differently to BA supplementation. However, little investigation has been conducted on ergogenic effects of BA in female team sport players. Twenty-eight-day BA administration improved peak torque and work completed by female masters’ athletes, but had no effect on body composition (Glenn et al., 2016). In contrast, 28-day BA supplementation had no significant effect on aerobic and anaerobic exercise capacity in recreationally active females. It has been suggested that trained muscles are more responsive to BA ingestion (Bex et al., 2014). In addition to buffering potential, *in vivo* studies have proposed antioxidant properties for carnosine (Kohen et al., 1988) i.e., exerting antioxidant effect scavenging reactive oxygen species and preventing oxidative damage (Gariballa and Sinclair, 2000). Nagasava et al. (2001) suggested that muscle carnosine exhibited a protective role against protein and lipid oxidation under oxidative stress conditions. However, this has not been confirmed in a real-life setting with overproduction of oxidative markers such as during exhaustive exercise.

Therefore, in the present study we investigated the effect of 4-week BA supplementation on basketball performance, aerobic, anaerobic and intermittent exercise performance and lactate concentration in collegiate female basketball players. We examined the effect of BA ingestion on the markers of muscle damage including malondialdehyde (MDA), creatine kinase (CK) and lactate dehydrogenase (LDH), as well.

## Methods

### Participants

Twenty-two female basketball players (mean ± SD, age: 21.7 ± 1.2 y, body mass: 63.2 ± 4.1 kg, height 167.0 ± 5.6 cm, *V̇O*_2max_ 43.9 ± 3.2 ml·kg^-1^·min^- 1^) volunteered to participate in the study. Participants were collegiate basketball players who typically underwent 6-8 h of training per week over 5 years. They completed four conditioning sessions per week throughout the study. All participants were non-smokers and had not used dietary supplements during the previous 6 months. The study procedure complied with the principles of the *Declaration of Helsinki* for human research and was approved by the institutional ethics committee. Participants were informed about the study procedures before they provided written consent.

### Design and Procedures

We used a randomized double-blind placebo-controlled design. Initially, anthropometric measurements were performed and maximal aerobic capacity (*V̇O*_2max_) was assessed using indirect calorimetry (MetaMax 3B, Cortex, CPET Germany). Participants were then randomly divided into BA and placebo supplementation groups, with stratification based on their playing position (forward, center and guard). Participants were assessed for aerobic, anaerobic, intermittent exercise performance and basketball skills before and after the supplementation period (Figure 1). All performance assessments were conducted at a similar external temperature (20 - 22°C). Blood samples were collected before and after the exhaustive treadmill test. Participants were required to abstain from caffeine and any sports supplements during the experimental period and from strenuous exercise during the 48 h preceding each trial. Since all participants were housed in the same place, they received a similar diet throughout the duration of the study. They were instructed to record their food intake throughout the 24-h period preceding the first trial and to replicate the same food and fluid intake prior to the second trial. Additionally, they were encouraged to follow a constant sleep timetable at a minimum of seven hours throughout the study duration.

### Supplementation protocol

BA or a placebo (dextrose) were provided to participants in similar capsules in order to mask the supplement and the placebo. The BA group consumed 6.4 g·day^-1^ of BA and the control group ingested isocaloric placebo ( (dextrose). Participants were instructed to consume 6.4 g·day^-1^ of BA in eight daily doses of 800 mg to avoid any overdose-related side effect such as paresthesia. They consumed two capsules immediately before and following exercise sessions and the remaining were consumed throughout the day. Compliance with the supplementation throughout the study was checked by the participants' consumption diaries.

### Measurements

#### Performance assessments

Participants completed an incremental running test to exhaustion on a treadmill (H/P/Cosmos 30000va08, Mercury/ Med, Germany) and underwent a standard Bruce protocol to assess aerobic capacity (Hanson et al., 2016). They were fitted with a mouthpiece for the measurement of oxygen uptake and a chest strap for heart rate monitoring. The test began with 1.7 m·h^-1^ and a 10% incline which increased at each stage every 3-min until volitional exhaustion. Anaerobic power was assessed using the 30-s Wingate test on a cycle ergometer (Monark, Ergomedic 894E, Healthcare International). The test consisted of pedaling at maximum speed against the determined load according to each participant's body mass (0.086 kg·kg^-1^ body mass). During the test strong verbal motivation was given to participants. Seat height was adjusted to each participant’s height and this height was recorded to be replicated in the post-intervention session.

The Yo-Yo test (Hoffman et al., 2008) was used to assess intermittent exercise performance. Participants completed two 20-m shuttle runs with 180° changes of directions and with 5 m of active recovery between each 20-m run. In this test a progressive incremental speed was controlled by an audio system. Exhaustion was assumed when the participant twice consecutively failed to touch the finish line. The total distance covered plus the incomplete final run was considered as the overall performance score. Countermovement jump records were measured by a force plate (Kistler 9284, Kistler instrument AG, Winterthur, Switzerland) and the records of free throw shots were assessed on a wooden indoor basketball court. The best jumping height from three efforts was considered as the jump record. A set of 30 free throw shots was performed by participants and the efficacy was evaluated by the total free throw shots performed.

It should be noted that a 15-min warm up and a 10-min cool down, comprising brisk walking/jogging, pedaling at a slower pace and stretching, were included in each testing session.

#### Anthropometry

Before and after the intervention period body composition was assessed using a bioelectrical impedance analyzer (Inbody230, Inbody Co, Seoul, Korea) in a fasting state. The measurements were performed with participants wearing minimal clothing and without shoes and socks. Height was measured without shoes to the nearest 0.5 cm.

#### Blood sample analysis

Blood samples were taken by an indwelling cannula (inserted into an antecubital vein) before and following the exhaustive exercise test at pre- and post-test sessions. Blood samples were transferred into EDTA-containing tubes and then were centrifuged at 3,000 rpm for 10 min to separate plasma samples for analysis of lactate concentration and LDH, CK and MDA activity. Plasma lactate concentration was measured by a photometric quantitative assay with intra-assay and inter-assay precision of 1.15% and 1.16%, respectively (Pars Azmoon, Iran). LDH was assessed by a quantitative photometric assay with intra-assay and inter-assay precision of 1.69% and 1.26%, respectively (Pars Azmoon, Iran). CK was also measured by a quantitative photometric assay with intra-assay and inter-assay precision of 0.52% and 0.53%, respectively (Pars Azmoon, Iran). MDA was measured by a colorimetric method with intra-assay and inter-assay precision of 5.8% and 7.6%, respectively (ZellBio, Germany).

#### Statistical analysis

Data were analyzed using SPSS for Windows version 25 (SPSS Inc, Chicago, III). Normal distribution of the data was confirmed by the Shapiro-Wilk test. Significant differences between groups were assessed using two-way analysis of variance (ANOVA). A paired *t*-test was also used to compare pre- and post-test values and an independent *t*-test was applied to detect intergroup differences at baseline. Data are expressed as mean ± SD and statistical significance was set at *p* < 0.05.

## Results

No side effects related to BA supplementation were reported throughout the study. Mean values and standard deviation of body composition components including body mass, BMI and fat% are indicated in Table 1. No significant differences at baseline levels were identified (all *p* > 0.05). Since participants followed a similar training program, training loads did not differ between groups throughout the study duration.

**Table 1:** Mean ± SD of anthropometric measures and V̇ O_2__max_ before and after the intervention.

Variable	BA (n=11)		Placebo (n=11)	
	
	Pre	Post	Pre	Post
Body mass (kg)	62.1 ± 5.2	61.8 ± 4.3	64.4 ± 5.0	63.8 ± 4.6
BMI (kg·m^-2^)	23.1 ± 2.6	23.0 ± 2.4	23.6 ± 2.1	23.5 ± 2.1
Fat (%)	18.1 ± 3.1	17.9 ± 2.5	19.3 ± 2.4	19.1 ± 2.6
VȮ_2max_ (ml·min^-1^·kg^-1^)	43.3 ± 4.7	46.1 ± 5.4*	44.5 ± 5.1	46.4 ± 4.9

*BMI; body mass index, V̇O*_*2max*_; *maximum oxygen uptake*.

* *Significant difference between pre- and post-test values (p < 0.05)*.

### Blood Variables

Lactate concentration increased following exercise at both pre- and post-test conditions for both groups (all *p* < 0.05), but the peak of exercise-following supplementation in the BA compared to the placebo group (*p* = 0.001; Table 2).

**Table 2:** Mean ± SD of blood variables in response to exhaustive exercise before and following the 4-week placebo and beta-alanine supplementation.

Variable	BA (n=11)				Placebo (n=11)					
	
	Baseline		Post-supplementation		Baseline		Post-supplementation		*p*	ES
	
	Pre-Exc	Post-Exc	Pre-Exc	Post-Exc	Pre-Exc	Post-Exc	Pre-Exc	Post-Exc		
Lactate (mmol/6)	2.2 ± 0.3	8.1 ± 0.7*	2.0 ± 0.3	6.2 ± 0.5*	2.0 ± 0.3	7.6 ± 8.9*	2.0 ± 0.2	7.0 ± 0.8*	0.01^#^	0.2 58
LDH (U·L^-1^)	346.3 ± 24.5	467.2 ± 34.4*	334.3 ± 27.5	451.2 ± 29.6*	369.2 ± 33.3	487.5 ± 41.0*	365.3 ± 28.5	478.2 ± 42.1*	0.24	0.0 72
CK (U·L^-1^)	78.2 ± 11.6	126.4 ± 16.1*	75.3 ± 12.1	119.8 ± 18.7*	86.3 ± 13.2	130.5 ± 20.6*	89.2 ± 12.1	137.5 ± 21.3*	0.40	0.0 35
MDA (μM)	2.1 ± 0.3	5.3 ± 0.6	2.0 ± 0.3	5.1 ± 0.6	2.4 ± 0.4	5.6 ± 0.6	2.5 ± 0.4	6.1 ± 0.8	0.30	0.0 58

*ES; effect size, CK; creatine kinase, LDH; lactate dehydrogenase, MDA; malondialdehyde*.

* *Significant difference between pre- and post-test values (p < 0.05)*.

# *Significant time × group interaction (p < 0.05)*.

Regardless of supplement/placebo ingestion and pre/post-test measurements, plasma levels of LDH, CK and MDA increased in response to acute exhaustive exercise (all *p* < 0.05). However, there was no significant difference between groups regarding LDH, CK and MDA responses to exercise (*p* = 0.24, *p* = 0.40 and *p* = 0.30, respectively; Table 2).

#### Exercise Performance

Exercise performance variables are presented in Table 3. Data analysis indicated that regardless of supplement/placebo ingestion all performance measures including TTE (*p* = 0.001, *p* = 0.001, respectively for BA and placebo groups), peak power (*p* = 0.001 and *p* = 0.001, respectively for BA and placebo groups), minimum power (*p* = 0.001 and *p* = 0.001, respectively for BA and placebo groups), total distance covered in the Yo-Yo test (*p* = 0.001 and *p* = 0.01, respectively for BA and placebo groups), countermovement jump (*p* = 0.002 and *p* = 0.02, respectively for BA and placebo groups) and free throw shots (*p* = 001 and *p* = 003, respectively for BA and placebo groups) improved from pre- to post-test for both groups. Regarding inter-group differences, data analysis revealed no significant interaction between groups (*p* = 0.31, *p* = 0.28, *p* = 0.30, *p* = 0.73, *p* = 0.31, and *p* = 0.51, respectively) indicating no significant difference between them.

**Table 3:** Mean ± SD of performance measures before and following the 4-week placebo and beta-alanine supplementation.

Variable	*BA (n=11)*		*Placebo (n=11)*			
	
	Pre	Post	Pre	Post	*p*	ES
TTE (s)	646 ± 51	695 ± 47*	663 ± 62	699 ± 64*	0.31	0.053
Yo-Yo (m)	1316 ± 75	1350 ± 60*	1336 ± 76	1360 ± 70*	0.73	0.006
Peak power (W·kg^-1^)	6.1 ± 1.1	7.7 ± 0.9*	6.2 ± 0.7	7.3 ± 0.5*	0.28	0.059
Minimum Power (W·kg^-1^)	2.1 ± 0.4	3.2 ± 0.5	2.3 ± 0.3	3.1 ± 0.4	0.30	0.055
CMJ (cm)	32.2 ± 2.3	34.1 ± 1.7*	33.0 ± 1.9	34.0 ± 1.6*	0.31	0.051
Free Throw Shots	17.9 ± 2.3	18.7 ± 1.6*	18.0 ± 1.9	19.1 ± 1.2*	0.51	0.015

*ES; effect size, CMJ; countermovement jump, TTE; time to exhaustion*.

* *Significant difference between pre- and post-test values (p < 0.05)*.

## Discussion

The main finding of the present study was that BA consumption significantly lowered lactate accumulation after exhaustive exercise in collegiate female basketball players; however, there was no significant effect on muscle damage indices and performance measures over a period of four weeks.

### Blood Variables

Lactate accumulation in response to exercise was significantly reduced following BA consumption. Chung et al. (2012) showed a small effect of BA supplementation on peak lactate concentration. It has been previously established that BA augments cellular buffering capacity by increasing carnosine levels (Harris et al., 2006). The elevated cellular carnosine concentration increases cellular buffering capacity and contributes to the reduced plasma concentration levels after exhaustive exercise. Although lactate is not the cause of H^+^ accumulation, it is a reliable marker of metabolic acidosis and its production is accompanied by a pH decline (Robergs et al., 2004). It has been indicated that efflux of lactate to the blood circulation must be accompanied by H^+^ (Bishop et al., 2010). The reduction of plasma lactate accumulation following exhaustive exercise at post supplementation may be explained by the buffering effect of BA. Another reason could be that lactate concentration in the circulation is the sum of production and clearance. Thus, it might be assumed that BA administration improves mitochondrial adaptation which may result in less lactate and H^+^ production (Bishop et al., 2010).

Carnosine, abundant in skeletal muscle, has also been suggested to exert antioxidant activity and prevent lipid peroxidation and protein damage (Guiotto et al., 2005). Nagasawa et al. (2001) indicated that carnosine inhibited lipid peroxidation and oxidative damage to proteins. They evaluated lipid peroxidation by thiobarbituric acid reactive substances (TBARS) and observed that carnosine reduced the TBARS level induced by the Fenton reactant (Fe, H_2_O_2_). Therefore, we hypothesized that BA might elevate antioxidant defense and could attenuate oxidative damage to muscle cells and lipid peroxidation in response to exhaustive exercise. However, four weeks of BA supplementation had no significant effect on CK, LDH and MDA activity in collegiate female basketball players. This is the first study investigating the protective effect of BA administration on cell damage and lipid peroxidation indices in sports. However, the outcomes of previous *in vitro* studies may differ with what we observed in a real field study. In order to manifest potential protective effects of BA in real-world setting, a supplementation protocol other than what is being implemented for buffering effects may be required. Further research is warranted to clarify the antioxidative potential of BA supplementation in sports.

### Exercise Performance

All exercise performance measures improved from pre- to post-test regardless of supplement/placebo consumption. The improvement in exercise performance must be attributed to a structured training program over four weeks. We observed that BA supplementation had no significant impact on TTE and Wingate test results. Our findings are in line with some previous studies which have examined the effect of BA supplementation on different exercise modalities (Hoffman et al., 2008; Saunders et al., 2012; Sweeney et al., 2010). Hill et al. (2007) reported no effect of BA supplementation on fatigue index, peak power and mean power output during three repeated 30-s maximal cycle sprints. It has been suggested that performance is not affected by muscle buffering during exercise lasting less than 60 s (Bogdanis et al., 1996) and would therefore not be influenced by elevated levels of muscle carnosine resulting from BA supplementation (Hobson et al., 2012). However, some studies with longer duration exercise protocols were also not able to induce ergogenic effects of BA. Ducker et al. (2013) reported that BA supplementation over four weeks had no significant effect on 2000 m rowing performance in well-trained male rowers. Chung et al. (2012) also reported no effect of BA administration over ten weeks on swimming performance in male and female swimmers. We also hypothesized that BA might improve intermittent exercise performance and attenuate exercise-induced fatigue and result in a better physiological condition under which players could perform better. However, BA supplementation did not exert any significant ergogenic effect on intermittent exercise, CMJ and free throw shot performance which is in line with previous results on sprint (Milioni et al., 2012; Saunders et al., 2012; Sweeney et al., 2010) and technical performance (Hoffman et al., 2008; Milioni et al., 2012) in elite and non-elite athletes. Yet, this finding is in contrast with Saunders et al. (2012b) who reported an improvement in Yo-Yo intermittent exercise test results following 12-week BA supplementation in amateur soccer players. The discrepancy in outcomes might be explained by the type of exercise test and differences in participants. The intermittent exercise test employed by Saunders et al. (2012b) was the Yo-Yo IR2 which initiates at a higher speed (11.5 km·h^-1^), and may require higher demand of energy supply from the anaerobic glycolytic pathway. Saunders et al. (2012b) suggested that any improvement in exercise performance could be due to increased muscle buffering capacity with carnosine. Although we did not assess muscle carnosine content in the present study, it does not mean that we cannot confirm the effect of BA supplementation on muscle carnosine concentration since BA supplementation has been established to increase muscle carnosine concentration (Sale et al., 2010). A relationship between BA consumption and muscle carnosine content has been established. It has been suggested that the relative, but not absolute, increase in muscle carnosine is higher in females (Stegen et al., 2014), raising the question whether a higher relative increase in carnosine after BA consumption in females is relevant from a practical view in athletes. We assume that this dosing protocol would have increased carnosine content according to the relationship between the BA dose and carnosine content (Kendrick et al., 2009) as our dosing (6.4 g·day^-1^) was equal or higher than in previous studies. Thus, a lack of elevated muscle carnosine concentration in response to BA supplementation is less likely to explain the lack of an effect on exercise performance in this study. Based on the results of the study, BA may have trivial and non-significant ergogenic effects on exercise performance measures in collegiate female basketball players. Nevertheless, further research is required to clarify how sex differences can alter the ergogenic effects of BA in athletes.

The consumption of 6.4 g·day^-1^ BA over four weeks significantly reduced lactate accumulation during exhaustive exercise. However, it did not exert any significant effect on aerobic, anaerobic, intermittent exercise performance and specific basketball performance in female basketball players. In addition, BA supplementation had no significant effect on lipid peroxidation and indices of cell damage. Although, *in vitro* studies support protective effects of carnosine against oxidative damage, usual dosing of BA supplementation with 6.4 g·day^-1^ does not seem to be sufficient to exert protective effects on lipid peroxidation and cell damage in practice.

## Limitations

We did not include a correction for changes in plasma volume in response to exercise. However, since the main objective was to determine the difference between BA and placebo conditions, both of which followed the same procedure, we speculate that it is less likely to affect the main findings regarding the difference between BA and placebo treatment on lactate response to exercise.

## References

[j_hukin-2022-0034_ref_001] Baguet A., Reyngoudt H., Pottier A., Everaert I., Callens S., Achten E., Derave W. (2009). Carnosine loading and washout in human skeletal muscles. Journal of Applied Physiology.

[j_hukin-2022-0034_ref_002] Bex T., Chung W., Baguet A., Stegen S., Stautemas J., Achten E., Derave W. (2014). Muscle carnosine loading by beta-alanine supplementation is more pronounced in trained vs. untrained muscles. Journal of Applied Physiology.

[j_hukin-2022-0034_ref_003] Bishop D., Edge J., Davis C., Goodman C. (2004). Induced metabolic alkalosis affects muscle metabolism and repeated-sprint ability. Medicine and Science in Sports and Exercise.

[j_hukin-2022-0034_ref_004] Bishop D. J., Thomas C., Moore-Morris T., Tonkonogi M., Sahlin K., Mercier J. (2010). Sodium bicarbonate ingestion prior to training improves mitochondrial adaptations in rats. American Journal of Physiology-Endocrinology and Metabolism.

[j_hukin-2022-0034_ref_005] Bjelica D, Masanovic B, Krivokapic D. (2020). A comparative study of anthropometric measurements and body composition between junior football and basketball players from the Serbian National League. Baltic Journal of Health and Physical Activity.

[j_hukin-2022-0034_ref_006] Bogdanis G. C., Nevill M. E., Boobis L. H., Lakomy H. (1996). Contribution of phosphocreatine and aerobic metabolism to energy supply during repeated sprint exercise. Journal of Applied Physiology.

[j_hukin-2022-0034_ref_007] Chung W., Shaw G., Anderson M. E., Pyne D. B., Saunders P. U., Bishop D. J., Burke L. M. (2012). Effect of 10 week beta-alanine supplementation on competition and training performance in elite swimmers. Nutrients.

[j_hukin-2022-0034_ref_008] Derave W., Ozdemir M. S., Harris R. C., Pottier A., Reyngoudt H., Koppo K., Wise J. A., Achten E. (2007). β-Alanine supplementation augments muscle carnosine content and attenuates fatigue during repeated isokinetic contraction bouts in trained sprinters. Journal of Applied Physiology.

[j_hukin-2022-0034_ref_009] Ducker K. J., Dawson B., Wallman K. E. (2013). Effect of Beta-alanine supplementation on 2,000-m rowing-ergometer performance. International Journal of Sport Nutrition and Exercise Metabolism.

[j_hukin-2022-0034_ref_010] Everaert I., Mooyaart A., Baguet A., Zutinic A., Baelde H., Achten E., Taes Y., De Heer E., Derave W. (2011). Vegetarianism, female gender and increasing age, but not CNDP1 genotype, are associated with reduced muscle carnosine levels in humans. Amino Acids.

[j_hukin-2022-0034_ref_011] Gariballa S. E., Sinclair A. J. (2000). Carnosine: physiological properties and therapeutic potential. Age and Ageing.

[j_hukin-2022-0034_ref_012] Glenn J. M., Gray M., Stewart Jr R. W., Moyen N. E., Kavouras S. A., DiBrezzo R., Turner R., Baum J. I., Stone M. S. (2016). Effects of 28-day beta-alanine supplementation on isokinetic exercise performance and body composition in female masters athletes. Journal of Strength & Conditioning Research.

[j_hukin-2022-0034_ref_013] Guiotto A., Calderan A., Ruzza P., Borin G. (2005). Carnosine and carnosine-related antioxidants: a review. Current Medicinal Chemistry.

[j_hukin-2022-0034_ref_014] Hanson N. J., Scheadler C. M., Lee T. L., Neuenfeldt N. C., Michael T. J., Miller M. G. (2016). Modality determines VO 2max achieved in self-paced exercise tests: validation with the Bruce protocol. European Journal of Applied Physiology.

[j_hukin-2022-0034_ref_015] Harris R. C., Tallon M., Dunnett M., Boobis L., Coakley J., Kim H. J., Fallowfield J. L., Hill C., Sale C., Wise J. A. (2006). The absorption of orally supplied β-alanine and its effect on muscle carnosine synthesis in human vastus lateralis. Amino Acids.

[j_hukin-2022-0034_ref_016] Hill C., Harris R. C., Kim H., Harris B., Sale C., Boobis L., Kim C., Wise J. A. (2007). Influence of β-alanine supplementation on skeletal muscle carnosine concentrations and high intensity cycling capacity. Amino Acids.

[j_hukin-2022-0034_ref_017] Hobson R. M., Saunders B., Ball G., Harris R., Sale C. (2012). Effects of β-alanine supplementation on exercise performance: a meta-analysis. Amino Acids.

[j_hukin-2022-0034_ref_018] Hoffman J. R., Ratamess N. A., Faigenbaum A. D., Ross R., Kang J., Stout J. R., Wise J. A. (2008). Short-duration β-alanine supplementation increases training volume and reduces subjective feelings of fatigue in college football players. Nutrition Research.

[j_hukin-2022-0034_ref_019] Kendrick I. P., Kim H. J., Harris R. C., Kim C. K., Dang V. H., Lam T. Q., Bui T. T., Wise J. A. (2009). The effect of 4 weeks β-alanine supplementation and isokinetic training on carnosine concentrations in type I and II human skeletal muscle fibres. European Journal of Applied Physiology.

[j_hukin-2022-0034_ref_020] Kohen R., Yamamoto Y., Cundy K. C., Ames B. N. (1988). Antioxidant activity of carnosine, homocarnosine, and anserine present in muscle and brain. Proceedings of the National Academy of Sciences.

[j_hukin-2022-0034_ref_021] Lyons M., Al-Nakeeb Y., Nevill A. (2006). The impact of moderate and high intensity total body fatigue on passing accuracy in expert and novice basketball players. Journal of Sports Science & Medicine.

[j_hukin-2022-0034_ref_022] Milioni F., Redkva P. E., Barbieri F. A., Zagatto A. M. (2017). Six weeks of β-alanine supplementation did not enhance repeated-sprint ability or technical performances in young elite basketball players. Nutrition and Health.

[j_hukin-2022-0034_ref_023] Nagasawa T., Yonekura T., Nishizawa N., Kitts D. D. (2001). In vitro and in vivo inhibition of muscle lipid and protein oxidation by carnosine. Molecular and Cellular Biochemistry.

[j_hukin-2022-0034_ref_024] Padulo J., Laffaye G., Haddad M., Chaouachi A., Attene G., Migliaccio G. M., Chamari K., Pizzolato F. (2015). Repeated sprint ability in young basketball players: one vs. two changes of direction (Part 1). Journal of Sports Sciences.

[j_hukin-2022-0034_ref_025] Robergs R. A., Ghiasvand F., Parker D. (2004). Biochemistry of exercise-induced metabolic acidosis. American Journal of Physiology-Regulatory, Integrative and Comparative Physiology.

[j_hukin-2022-0034_ref_026] Sale C., Saunders B., Harris R. C. (2010). Effect of beta-alanine supplementation on muscle carnosine concentrations and exercise performance. Amino Acids.

[j_hukin-2022-0034_ref_027] Saunders B., Sale C., Harris R. C., Sunderland C. (2012). Effect of beta-alanine supplementation on repeated sprint performance during the Loughborough Intermittent Shuttle Test. Amino Acids.

[j_hukin-2022-0034_ref_028] Saunders B., Sunderland C., Harris R. C., Sale C. (2012). β-alanine supplementation improves YoYo intermittent recovery test performance. Journal of the International Society of Sports Nutrition.

[j_hukin-2022-0034_ref_029] Stegen S., Bex T., Vervaet C., Vanhee L., Achten E., Derave W. (2014). β-Alanine dose for maintaining moderately elevated muscle carnosine levels. Medicine and Science in Sports and Exercise.

[j_hukin-2022-0034_ref_030] Sweeney K. M., Wright G. A., Brice A. G., Doberstein S. T. (2010). The effect of β-alanine supplementation on power performance during repeated sprint activity. Journal of Strength & Conditioning Research.

